# “Market withdrawals” of medicines in Germany after AMNOG: a comparison of HTA ratings and clinical guideline recommendations

**DOI:** 10.1186/s13561-018-0209-3

**Published:** 2018-09-18

**Authors:** Thomas R. Staab, Miriam Walter, Sonja Mariotti Nesurini, Charalabos-Markos Dintsios, J.-Matthias Graf von der Schulenburg, Volker E. Amelung, Jörg Ruof

**Affiliations:** 1 Roche Pharma AG, Grenzach-Wyhlen, Germany; 2 nspm ltd, Meggen, Switzerland; 3 Health Services Research and Health Economics, Heinrich Heine University, Düsseldorf, Germany; 4 Leibniz University Hanover, Hanover, Germany; 5 Medical School of Hanover, Hanover, Germany; 6 r-connect ltd, Hauensteinstr. 132, 4059 Basel, Switzerland

**Keywords:** AMNOG, Early benefit assessment, Product recalls and withdrawals, Opt-out

## Abstract

**Background:**

According to the AMNOG act, the German Federal Joint Committee (G-BA) determines the additional benefit of new medicines as a basis for subsequent price negotiations. Pharmaceutical companies may withdraw their medications from the market at any time during the process. This analysis aims to compare recommendations in clinical guidelines and HTA appraisals of medicines that were withdrawn from the German market since the introduction of AMNOG in 2011.

**Methods:**

Medications withdrawn from the German market between January 2011 and June 2016 following benefit assessment were categorized as opt-outs (max. 2 weeks after start of price negotiations) or supply terminations (during or after further price negotiations). Related guidelines were systematically analyzed. For all withdrawals, therapeutic area, additional benefit rating and recommendation status in relevant clinical guidelines were assessed.

**Results:**

Among 139 medications, 10 opt-outs and 12 supply terminations were identified. Twenty-one out of 22 withdrawn medicines (95%) received ‘no additional benefit’ appraisal by the G-BA (average ‘no additional benefit’ rating for all AMNOG products: 47%). Of the 22 medicines, 15 (68%) were recommended by at least one guideline at the time of benefit assessment and 18 (82%) on 1 June 2016. Heterogeneity among guidelines was high. Acceptance of clinical trial endpoints was different between G-BA appraisals and clinical guidelines.

**Conclusion:**

Our analysis revealed considerable differences across clinical guidelines as well as between clinical guidelines and HTA appraisals of the medicines that were withdrawn from the German market. Better alignment of the clinical perspective and close collaboration between all involved parties is required to achieve and maintain optimization of patient care.

**Electronic supplementary material:**

The online version of this article (10.1186/s13561-018-0209-3) contains supplementary material, which is available to authorized users.

## Background

Health technology assessments (HTA) of innovative medicines are a common feature all across Europe. A key challenge for all healthcare systems is how to decide which medicines should be covered under the national reimbursement scheme. In the United Kingdom, a predefined cost-effectiveness ratio determines a threshold for reimbursement [1]. In France, the SMR (‘service médical rendu’, actual clinical benefit) and ASMR (‘amélioration du service médical rendu’, improvement in actual clinical benefit) determine the price level and the rate of reimbursement by the national health insurance [2].

In Germany, the manufacturer has to submit a benefit dossier at the time of market entry. Thereafter, the IQWIG (‘Institut für Qualität und Wirschaftlichkeit im Gesundheitswesen’) reviews the dossier before the Federal Joint Committee (‘Gemeinsamer Bundesausschuss’, G-BA) conducts the appraisal of the additional benefit of the innovative medicine versus the current standard of care. Based on the outcome of the benefit appraisal, the National Association of the Statutory Health Insurance Funds (‘GKV Spitzenverband’, GKV-SV) and the manufacturer enter into price negotiations. A mutually agreed price is in place 1 year after market entry. During the initial 12 months after market entry, the medication is sold at a price set by the manufacturer, i.e. newly licensed drugs are available without restriction in the German health care system as soon as they enter the German market. Should the parties not come to an agreement on the sales discount, an arbitration board is called in. The legal basis for this procedure is anchored in the Act on the Reform of the Market for Medical Products (‘Arzneimittelmarktneuordnungsgesetz’, AMNOG), introduced in 2011 [3–5].

The manufacturer has the legal right to resign from price negotiations at the latest 2 weeks after the first negotiation meeting; this is referred to as opt-out. Termination of supplies can be chosen as a second option either before, during or after price negotiations. In both cases, the medicine is then withdrawn from the German market [6]. Although after official withdrawal of a specific medicine from the German market, patients may be able to continue receiving it via individual imports, this is usually associated with high administrative effort [7].

A particular challenge to any HTA body are accelerated approval procedures for innovative medicines that are applied both in the US and in Europe [8]. Available data at the time of market entry are often immature, making the assessment of the additional benefit difficult.

It has been shown that in clinical practice, the possibility of a medicine being withdrawn has a major impact on individual decision making. This is particularly relevant in the context of AMNOG: a German federal state association of panel doctors urged their members to account for the possibility of market withdrawals when prescribing newly approved medicines for which no reimbursed price had been agreed on yet [9]. In line with this, in a survey of 150 German physicians, 67% reported considering the possibility of market withdrawal when making therapeutic decisions. This indicates that physicians may be hesitant to initiate therapies with novel medicines because these might be withdrawn from the market and a therapy change would be required in all patients receiving them [10].

While HTA procedures are mandatory and fully justified in an environment of steadily increasing health care costs, the respective appraisals, subsequent price negotiations, and optional withdrawal decisions by the pharmaceutical manufacturers should not lead to a deterioration of treatment options for patients. It is the authors’ position that medicines that are recommended in clinical guidelines should be available as an option for treating patients.

Our analysis therefore aims to compare clinical guideline recommendations and HTA appraisals of medicines that were withdrawn from the German market.

## Methods

### Analysis set

Opt-outs and supply terminations since introduction of the benefit assessment in 1 January 2011 up until 1 June 2016 were identified in the AMNOG Report, the GKV-SV database, the German medicine atlas, the German prescription report and by manual search [10–13].

Up until January 2014, when changes in policy were put in place, the G-BA also assessed the benefit of medications which had already been on the German market. This assessment of the existing market covered therapeutic areas of chronic pain, osteoporosis, cardiovascular diseases, and diabetes mellitus [14]. Market withdrawals from both early benefit assessments and assessments of the existing markets were taken into account. Medicines that were temporarily withdrawn from the market but reintroduced before June 1st 2016 were reviewed but excluded from the systematic analysis. Products that changed their brand name, were excluded from the analysis if supply of the molecule was continuously guaranteed throughout the observation time. Moreover, orphan medicines were reviewed but not included in the systematic analysis as the G-BA does not determine an appropriate comparative therapy for orphan medicines and therefore no systematic analysis of guidelines is performed.

### Therapeutic areas

All therapeutic areas were included. Medication assignment to therapeutic areas was done in line with the G-BA [15].

### Benefit assessments

Details on the benefit assessments including date of decision, extent of granted additional benefit, and reason for no additional benefit, if applicable, were obtained from the G-BA database [15]. In cases where the G-BA re-assessed certain medications in the same indication, only the latest benefit assessment was evaluated. As a conservative approach, if a drug was assessed in several patient groups, the best rating was used for the analysis. The reasons for not granting an additional benefit were considered in the following order (corresponding to decreasing levels of demonstrated clinical advantage): medicine judged as showing insufficient clinical superiority according to the G-BA appraisal, no appropriate data according to the G-BA appraisal (e.g. because an inappropriate comparator was used), or no dossier submitted. If several reasons were provided for a given drug due to several indications and/or subgroups, the first applicable reason (corresponding to the highest level of demonstrated clinical advantage) was considered.

### Arbitration procedures

Medications that underwent the arbitration procedure were extracted from a recent publication [5]. As this publication only included documents up to January 2016, a manual search for further procedures was conducted.

### Approval and withdrawal dates

The date of approval by the European Medicines Agency (EMA) was extracted from the database of European public assessment reports [16]. To assess the date of withdrawal, the latest entry in the German pharmaceutical catalogue, the *Lauer-Taxe*, was consulted [17].

### Guideline recommendations

The G-BA chooses the appropriate comparative therapy for the benefit assessment based on, among other factors, relevant literature and guidelines identified in a systematic literature search prior to the assessment (according to the G-BA Rules of Procedure, par. 7.2) [4].

Recommendation status at the time of benefit assessment was analyzed using the guidelines identified by the G-BA’s systematic literature search. Guidelines may recommend either ‘specific medicines’ or a ‘class’ of medicines only. Within our analysis we discriminated between those two levels of recommendations with the latter being considered the weaker level of recommendation. In cases where the G-BA re-assessed certain medications in the same indication, the earliest benefit assessment was evaluated. To assess recommendation status at the time of analysis (1 June 2016), guidelines were identified by a) a search for the version that was current on 1 June 2016 for all guidelines used by the G-BA, and b) a manual search, using the G-BA algorithm, for evidence-based guidelines newly published until 1 June 2016 (Additional file 1: Figure S1). If a guideline used by the G-BA was not available anymore, the information provided in the G-BA documentation was analyzed. Guidelines were analyzed in terms of the methodology applied, i.e. whether they included i) a systematic rating of evidence, which allowed for a ranking of recommendations, and ii) a graphical display of a suggested treatment algorithm. Country of origin was determined for each guideline, and for German guidelines, we also evaluated whether guidelines adhered to the S3 category, reflecting the highest methodological standard, according to the classification of the Association of the Scientific Medical Societies in Germany [18].

Recommendation status was assessed as follows:

- Medications, or their class, were defined as *recommended* if at least one of the identified guidelines issued a positive recommendation, i.e. if a medicine or class was either included in a recommendation or specifically mentioned as a valid treatment option in the text.
- If a guideline recommended the specific medicine as well as the class, this was counted as a *recommendation for the specific medicine* only.

## Results

### Analysis set

In total, 139 products were evaluated by the G-BA in 14 different therapeutic areas in the period between January 2011 and June 2016. Of these, 22 products (16%) were withdrawn from the market. Three additional products (bosutinib, dapagliflozin and pitavastatin) were only temporarily withdrawn from the market and were therefore not included in the full analysis:

- For pitavastatin, a medicine for the treatment of hypercholesterolemia, no dossier was submitted to the G-BA. The medicine was temporarily withdrawn from the market but reintroduced after a fixed reference price had been determined.
- Bosutinib, an orphan medicine for the treatment of chronic myelogenous leukemia, received a non-quantifiable benefit rating from the G-BA. During price negotiation, it was temporarily withdrawn from the market but reintroduced before finalization of the final rounds of price negotiation.
- Dapagliflozin, indicated for the treatment of diabetes, was not granted an additional benefit by the G-BA. It was temporarily withdrawn from the market but reintroduced after price agreement has been reached.

**Fig. 1:**
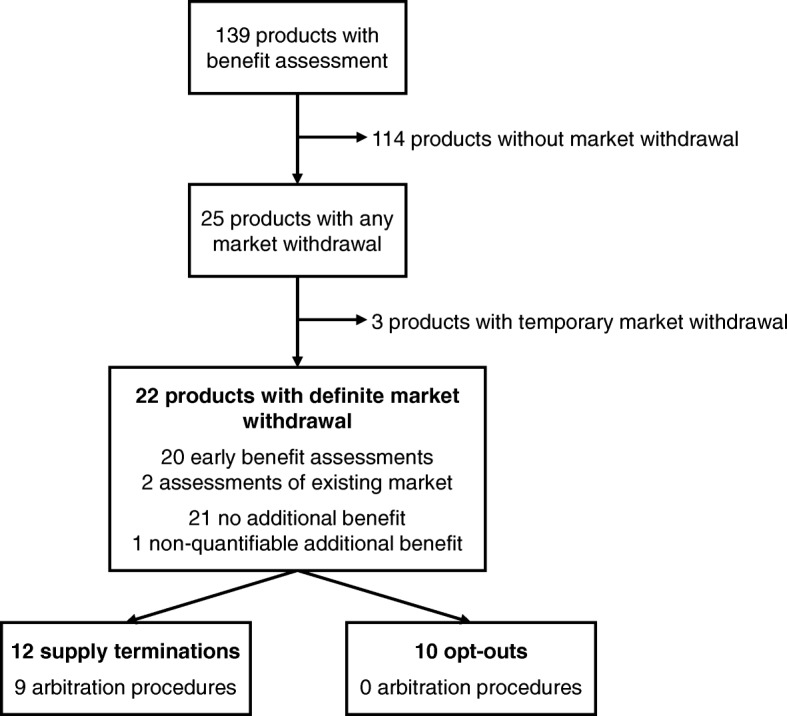
Dataset used for the analysis of market withdrawals

Figure 1 provides an overview of the complete analysis set.

Detailed information on the 22 withdrawn products is summarized in Table 1. Only 2 of the 22 medications, vildagliptin and vildagliptin/metformin, underwent an assessment of the existing market by the G-BA; all other 20 products passed through the early benefit assessment.

**Table 1:** Medications withdrawn from the German market since the introduction of AMNOG benefit assessments

Active ingredient (brand name)	Manufacturer	Therapeutic area	EMA approval	Unlisted^a^	Type of withdrawal	Arbitration procedure
Aliskiren/amlodipine (Rasilamlo)	Novartis Pharma	Cardiovascular	14 Apr 2011	1 Sep 2011	Opt-out	NA^b^
Bromfenac (Yellox)	Bausch & Lomb/Dr. Mann Pharma	Ophthalmic	18 May 2011	1 May 2014	Supply termination	Yes
Canagliflozin (Invokana)	Janssen-Cilag	Metabolic	15 Nov 2013	15 Oct 2014	Opt-out	NA^b^
Canagliflozin/metformin (Vokanamet)	Janssen-Cilag	Metabolic	23 Apr 2014	1 Mar 2015	Opt-out	NA^b^
Colestilan (BindRen)	Mitsubishi Pharma	Other	21 Jan 2013	1 Apr 2015	Supply termination	No^c^
Gaxilose (LacTest)	Venter Pharma S.L.	Metabolic	NA^d^	1 Mar 2016	Opt-out	NA^b^
Insulin degludec (Tresiba)	Novo Nordisk Pharma	Metabolic	21 Jan 2013	15 Jan 2016	Supply termination	Yes
Living larvae from *Lucilia sericata* (BioBag)	BioMonde GmbH	Other	NA^d^	15 Jun 2015	Supply termination^i^	Yes
Linaclotide (Constella)	Almirall Hermal	Digestive	26 Nov 2012	15 Jul 2014	Supply termination	Yes
Linagliptin (Trajenta)	Boehringer Ingelheim Pharma	Metabolic	24 Aug 2011	1 Jan 2012	Opt-out	NA^b^
Lixisenatide (Lyxumia)	Sanofi-Aventis Deutschland	Metabolic	1 Feb 2013	15 Jun 2014	Supply termination	Yes
Lomitapide (Lojuxta)	Aegerion Pharmaceuticals	Metabolic	31 Jul 2013	1 Aug 2014	Opt-out	NA^b^
Lurasidone (Latuda)	Takeda GmbH	Psychiatric	21 Mar 2014	15 Nov 2015	Supply termination	No
Microbial collagenase (Xiapex)	Pfizer Pharma	Musculoskeletal	28 Feb 2011	15 Jun 2012	Opt-out	NA^b^
Mirabegron (Betmiga)	Astellas Pharma GmbH	Genitourinary	20 Dec 2012	1 Jun 2015	Supply termination	Yes
Perampanel (Fycompa)	Eisai	CNS	23 Jul 2012	1 Aug 2014	Supply termination	Yes
Regorafenib (Stivarga)	Bayer Vital	Oncology	26 Aug 2013	15 May 2016	Opt-out	NA^b^
Retigabine (Trobalt)	GlaxoSmithKline	CNS	28 Mar 2011	1 Jul 2012	Opt-out	NA^e^
Sipuleucel-T (Provenge)	Dendreon UK Limited	Oncology	6 Sep 2013	15 Jul 2015	Supply termination	No
Tafluprost/timolol (Taptiqom)	Santen	Ophthalmic	NA^d^	1 Aug 2015	Opt-out	NA^b^
Vildagliptin^f^ (Galvus, Jalra, Xiliarx)	Novartis Pharma	Metabolic	26 Sep 2007	15 Sep 2014 1 Jul 2014^g^	Supply termination	Yes
Vildagliptin/metformin^f^ (Eucreas, Icandra, Zomarist)	Novartis Pharma	Metabolic	14 Nov 2007	15 Sep 2014 1 Jul 2014^h^	Supply termination	Yes

^a^Date the medication was removed from the German pharmaceutical catalogue (Lauer-Taxe), which is updated bi-monthly

^b^Not applicable as opt-out medications do not enter price negotiations

^c^An arbitration procedure was initiated [42] but not completed [5]

^d^Not applicable as decentralized approval

^e^The manufacturer opted out before the price negotiations; however, price negotiations and eventually an arbitration procedure were subsequently initiated by a parallel importer [5]

^f^Assessment of the existing market

^g^For Galvus and Jalra, respectively (Xiliarx is marketed by foreign third parties)

^h^For Eucreas and Icandra, respectively (Zomarist is marketed by foreign third parties)

^i^Supply termination only for outpatient services, medicine still available in hospital settings

AMNOG: Act on the Reform of the Market for Medical Products; CNS: central nervous system; EMA: European Medicines Agency

Ataluren was the only orphan medicine within our sample. It is indicated for Duchenne muscle dystrophy, a rare disease with < 100 patients in Germany. A guideline review was not part of the G-BA assessment and ataluren was therefore not included in the analysis. Nivolumab in the indication ‘non-small cell lung cancer’ was initially introduced leveraging the brand name ‘Nivolumab BMS’. This brand was withdrawn from the market and marketing authorization shifted to ‘Opdivo’. As the molecule was continuously available for patients it was not included in our analysis.

For 10 medications (45%), the manufacturers opted out from entering price negotiations, and for 12 products (55%), the supply was terminated. For two medications (9%), supply was terminated not only in Germany, but across Europe: for both sipuleucel-T (Provenge), authorized for the treatment of prostate cancer, and colestilan (BindRen), authorized for the treatment of hyperphosphatemia in chronic kidney disease, the EMA (European Medicines Agency) withdrew marketing authorization at the request of the manufacturer for commercial reasons [19, 20]. Living larvae of *Lucilia sericata* were withdrawn from the outpatient market only and are still available in the hospital setting. Another product, retigabine, authorized for the treatment of drug-resistant partial onset epileptic seizures, has been discontinued worldwide by June 2017 due to limited usage [21]. Of the 12 medications that underwent supply terminations, 9 (75%) entered the arbitration procedure. For 9 of the 10 products with opt-out, there were no arbitration procedures as no price negotiations took place. For retigabine, price negotiations and an arbitration procedure were initiated by a parallel importer following the manufacturer’s opt-out [5]; however, the parallel importer never marketed the product either.

On average (± standard deviation) and excluding assessments of the existing market, opt-outs occurred 401 ± 271 days after marketing authorization (range: 130–993), whereas medications with supply termination had been available on the market for 747 ± 218 days (range: 489–1089).

### Therapeutic areas

Among the 139 medicines evaluated by the G-BA, the highest numbers of appraisals occurred in oncology (38 medicines) and metabolic disorders (30 medicines). In metabolic disorders, 9 out of 30 products (30%) were withdrawn from the market; in ophthalmic disorders, withdrawal rate was 33% (2 out of 6 medicines); in central nervous system disorders, it was 25% (2 out of 8 medicines); in cardiovascular diseases, 14% (1 out of 7 medicines); and in oncology, 5% (2 out of 38 medicines). No withdrawals occurred in the areas of infectious diseases (*N* = 16), respiratory diseases (*N* = 9), dermatology (*N* = 3), and hematology (*N* = 2), with *N* depicting the total number of assessed medications in these therapeutic areas.

### Benefit assessment

Additional benefit ratings for all withdrawn products are shown in Table 2. Although only the highest benefit rating was considered in the analysis if the G-BA assessed more than one subgroup and/or indication, 95% (21 out of 22) of the withdrawn medications received a ‘no additional benefit’ rating. The only remaining medication, sipuleucel-T, received an additional benefit rating of ‘not quantifiable’. The manufacturer had submitted data from three studies, which the G-BA determined as biased due to differences in post-progression therapies and the option of rescue therapy with a sipuleucel-T analogue specified in the protocol. The appraisal for sipuleucel-T was conditional and benefit assessment was scheduled to be repeated in April 2018 [22]. However, with the Europe-wide market withdrawal of sipuleucel-T, a reassessment of its benefit seems unlikely.

**Table 2:** Extent of additional benefit and recommendation status for all withdrawn medicines

Medicine	Reason for ‘no additional benefit’ rating^a^	Number of guidelines with positive recommendation (total number of guidelines reviewed)			
		At time of benefit assessment		Additional guidelines June 2016	
		Guidelines	Recommendation^b^	Guidelines	Recommendation^b^
Aliskiren/ amlodipine	No appropriate data	1 (4)	Medicine (aliskiren)	1 (2)	Medicine (aliskiren)
Bromfenac	No dossier submitted	4 (7)	Class (NSAID)	1 (2)	Class (NSAID)
Canagliflozin	Insufficient clinical superiority	1 (4)	Class (SGLT-2 inhibitors)	3 (3)	Medicine (canagliflozin)
Canagliflozin/ metformin	Insufficient clinical superiority	1 (5)	Class (SGLT-2 inhibitors/metformin)	3 (3)	Class (SGLT-2 inhibitors/metformin)
Colestilan	Insufficient clinical superiority	1 (1)	Class (phosphate binding agents)	1 (1)	Class (phosphate binding agents)
Gaxilose	No dossier submitted	n.a.	n.a.	n.a.	n.a.
Insulin degludec	Insufficient clinical superiority	7 (7)	Class (basal insulin analogues)	3 (3)	Medicine (insulin degludec)
Living larvae from *Lucilia sericata*	No dossier submitted	1 (1)	Medicine (living larvae)	n.a.	n.a.
Linaclotide	Insufficient clinical superiority	0 (4)	n.a.	1 (1)	Medicine (linaclotide)
Linagliptin	Insufficient clinical superiority	2 (3)	Class (DPP-4 inhibitors)	3 (3)	Medicine (linagliptin) and class (DPP-4 inhibitors)
Lixisenatide	Insufficient clinical superiority	5 (5)	Class (GLP-1 agonists)	3 (4)	Medicine (lixisenatide) and class (GLP-1 agonists)
Lomitapide	Insufficient clinical superiority	0 (2)	n.a.	1 (1)	Medicine (lomitapide)
Lurasidone	Insufficient clinical superiority	4 (4)	Medicine (lurasidone)	2 (2)	Class (second generation antipsychotic drugs)
Microbial collagenase	Insufficient clinical superiority	n.a.	n.a.	n.a.	n.a.
Mirabegron	Insufficient clinical superiority	0 (6)	n.a.	1 (1)	Medicine (mirabegron)
Perampanel	No appropriate data	0 (1)	n.a.	0 (1)	n.a.
Regorafenib	Insufficient clinical superiority	0 (7)	n.a.	3 (6)	Medicine (regorafenib)
Retigabine	No appropriate data	1 (1)	Medicine (retigabine)	1 (1)	Medicine (retigabine)
Sipuleucel-T	n.a.	4 (11)	Medicine (sipuleucel-T)	5 (9)	Medicine (sipuleucel-T)
Tafluprost/timolol	Insufficient clinical superiority	2 (2)	Class (preservative- free medicines)	n.a.	n.a.
Vildagliptin	Insufficient clinical superiority	5 (5)	Medicine (vildagliptin)	4 (4)	Medicine (vildagliptin) and class (DPP-4 inhibitor)
Vildagliptin/ metformin	Insufficient clinical superiority	5 (5)	Medicine (vildagliptin/metformin)	4 (4)	Class (DPP-4 inhibitors/metformin)

^a^All medicines had a ‘no additional benefit’ rating except Sipuleucel-T (‘non quantifiable benefit’)

^b^Recommendation of medicine or therapeutic class

DPP-4: dipeptidyl peptidase 4; GLP-1: glucagon-like peptide-1 receptor; n.a.: not applicable; NSAID: nonsteroidal anti-inflammatory drugs; SGLT-2: sodium-glucose co-transporter 2

Table 2 also displays the reasons why no additional benefit was granted (where applicable). For 15 of the 21 medications which were deemed without additional benefit, the G-BA determined that they demonstrated insufficient clinical superiority to the appropriate comparative therapy. Three dossiers did not report any appropriate data (aliskiren/amlodipine, retigabine, perampanel) and for three products, no dossier was submitted (bromfenac, gaxilose, living larvae of *Lucilia sericata*). Moreover, no appropriate data were reported for regorafenib in the second indication of gastrointestinal stromal tumor.

### Guideline recommendations

For all products, the guidelines taken into account by the G-BA upon initiation of the respective benefit assessment were evaluated to assess whether or not they recommended the withdrawn medication and/or its class at the time. To additionally determine the perception of the clinical value of the medication on 1 June 2016, the versions of the guidelines selected by the G-BA that were current on 1 June 2016, as well as newly published guidelines before this date, were also analyzed.

An overview of guidelines, their country of origin, the inclusion of an evidence rating scheme, the display of a graphical treatment algorithm, and their recommendations at the time of benefit assessment and on 1 June 2016 is provided in Additional file 2: Table S1. A total of 94 guidelines were reviewed. Thirty four guidelines covered oncological conditions (i.e. colorectal carcinoma, gastrointestinal stromal tumor, prostate cancer), 19 covered metabolic conditions (i.e. diabetes, hypercholesterolemia), 11 covered ophthalmic conditions (i.e. postoperative management of cataract surgery and glaucoma), 7 covered overactive bladder, 6 guidelines each covered hypertension and schizophrenia, 5 covered irritable bowel syndrome, 3 covered epilepsy, 2 covered hyperphosphatemia, and 1 covered wound healing.

Of those 94 guidelines, 82 (87%) were available as full publications. For the remaining guidelines, the G-BA documentation was analyzed as the documents were no longer available. Evidence ratings were applied by 72 (88%) of the 82 fully available guidelines. However, ratings were not consistent across guidelines. Graphically displayed treatment algorithms were available in 43 (52%) out of the 82 guidelines.

For gaxilose (metabolic diseases) and microbial collagenase (musculoskeletal disorders) no guidelines could be identified both by the G-BA and at the time of this analysis. For the benefit assessment of bromfenac (ophthalmic diseases), no systematic literature search was conducted by the G-BA. Instead, guidelines used for a German HTA rapid report in the relevant indication were used for this analysis [23].

Overall, 15 (68%) of the withdrawn medications had been recommended in at least one of the reviewed guidelines by name (*n* = 7; 32%) or class (*n* = 8; 36%) at the time of benefit assessment. Evaluation of the guidelines current as of 1 June 2016 showed an increase of overall recommended products to 18 (82%), of which 14 (64%) were recommended specifically and 4 (18%) by class. However, recommendation status of individual medicines remained inconsistent across guidelines and therapeutic classes (Table 3).

**Table 3:** Summary table of key issues by therapeutic area

Therapeutic area indication	Medicines	Key issues within therapeutic class
Cardiovascular		
Hypertension	Aliskiren/amlodipine	Aliskiren is recommended within clinical guidelines both as monotherapy and in combination with other antihypertensives. However, the fixed combination of aliskiren and amlodipine that was appraised by the G-BA is not covered within the guidelines
Ophthalmic		
Postoperative management of cataract surgery	Bromfenac	Guidelines suggest the therapeutic class (NSAID) in the perioperative period in cataract surgery, but do not specify any medicine
Glaucoma	Tafluprost/timolol	Guidelines strongly support the use of preservative-free medicines if there is evidence that patients are allergic to the preservative, but do not specify any medicine
Metabolic		
Diabetes	Canagliflozin Canagliflozin/metformin Linagliptine Lixisenatide Vildagliptine Vildagliptine/metformin	Guidelines evolved over time. While metformin remains the gold standard for initial drug therapy, guidelines support other classes and products such as canagliflozin and its class (SGLT-2 inhibitors), linagliptin and vildagliptin (DPP-4 inhibitors), and lixisenatide and its class (GLP-1 agonists) i) as monotherapy (SGLT-2 and DPP-4 inhibitors) in patients who are not eligible for initial metformin treatment and ii) as combination therapy (SGLT-2 and DPP-4 inhibitors and GLP-1 agonists)
	Insulin degludec	Basal insulin analogues are recommended within guidelines. Within that class, insulin degludec is one option
Hypercholesterolemia	Lomitapide	Lomitapide and other new therapeutic options are part of the suggested treatment algorithm in patients with homozygous familiar hypercholesterolemia
Digestive		
Irritable bowel syndrome	Linaclotide	Only one updated guideline is available [28]. This guideline recommends linaclotide as second-line treatment if previous laxatives did not help and patients had constipations for at least 12 months
Psychiatric		
Schizophrenia	Lurasidone	Guidelines generally recommend second generation antipsychotic drugs, but the evidence base for appropriate comparisons is considered limited
Musculoskeletal		
Dupuytren’s contracture	Microbial collagenase	Lack of relevant guidelines for the treatment of Dupuytren’s contracture
Genitourinary		
Overactive bladder	Mirabegron	Guidelines evolved over time and included mirabegron as second-line treatment [31]
CNS		
Epilepsy	Perampanel	Guidelines are heterogeneous [44] and partially not updated, e.g. the American Epilepsy Society is still presenting a 2004 publication on their homepage as guidance for refractory epilepsy.
	Retigabine	Retigabine is recommended as adjunctive second line treatment [32]
Oncology		
Colorectal carcinoma	Regorafenib	Regorafenib is recommended both in US and EU clinical guidelines [33] as second/third line of therapy.
Gastrointestinal stromal tumor	Regorafenib	Regorafenib is recommended as second/third line of therapy [34]
Prostate cancer	Sipuleucel-T	Sipuleucel-T is recommended by various guidelines in patients with metastatic prostate cancer and asymptomatic or minimally symptomatic disease
Other		
Hyperphosphatemia	Colestilan	Guidelines generally recommend phosphate binding agents but do not specify any medicine
Hypolactasia^a^	Gaxilose	No relevant guidelines were identified for hypolactasia.
Wound healing	Living larvae from *Lucilia sericata*	Only one guideline from 2012 is available [35]. Living larvae considered superior versus hydrogel therapy in terms of wound cleansing

^a^Hypolactasia was classified as a metabolic disorder by the G-BA

*DPP-4* dipeptidyl peptidase 4, *G-BA* Federal Joint Committee, *GLP-1* glucagon-like peptide-1 receptor, *NSAID* nonsteroidal anti-inflammatory drugs, *SGLT-2* sodium-glucose co-transporter 2

## Discussion

The AMNOG act was not introduced to control and manage the supply/use of medicines. Its aim was to manage prices and total public expenditure of pharmaceutical products [24]. In order to achieve this, a link between the reimbursed price of newly approved drugs and the additional benefit that they offer is stipulated. Clinical guidelines, on the other hand, are focused on treatment options for patients. Considering the different goals of G-BA appraisals and clinical guidelines, it is not necessarily contradictory that a medicine is recommended within a guideline while being assessed as having no additional benefit by the G-BA. However, several years after its coming into effect, it is becoming increasingly clear that the new legislation impacts the traditionally high availability of newly approved drugs on the German market due to opt-outs following benefit assessments [25], and the recent discussion about including G-BA appraisals into the physicians’ desk reference systems (‘Arztinformationssystem’) raises the question whether this may be to the disadvantage of patients and caregivers.

Our analysis showed that out of a total of 139 products evaluated by the G-BA between January 2011 and June 2016, 22 medicines (16%) were withdrawn from the German market. Twenty-one (95%) of those medicines received a ‘no additional benefit’ rating by the G-BA, a sharp contrast to the average of 43% of ‘no additional benefit ratings’ in all G-BA appraisals [10]. As both benefit appraisals and clinical guidelines rely on the principles of evidence-based medicine [26], those discrepancies are striking, raising the question of how these diverging evaluations of the same medicine fit together. The respective analysis reveals a couple of key features:

- An obvious heterogeneity between guideline recommendations and G-BA appraisals occurred in diabetes. The validity of HbA1C, a widely accepted primary endpoint in diabetes trials, is challenged by the G-BA [27], supporting the ‘no additional benefit’ decision by the G-BA for the majority of recently introduced diabetes drugs. The critical approach to widely accepted primary clinical trial outcomes by the G-BA is an example of a fundamental difference of German HTA appraisals and more clinically centered guidelines.
- Several of the 22 medicines such as linaclotide [28], lomitapide [29], lurasidone [30], mirabegron [31], retigabine [32], and regorafenib [33, 34] are recommended as later -line treatments in their respective disease area. While each of these medicines has to be considered individually, the unavailability of later-line treatment options generally carries a risk of suboptimal care, particularly in patients with advanced conditions.
- In some therapeutic areas, guidelines primarily support a class of products rather than specific medicines: nonsteroidal anti-inflammatory drugs are recommended for the postoperative management in cataract surgery, preservative-free medicines are supported for the subset of glaucoma patients allergic to preservatives, and phosphate binding medicines are recommended for the treatment of hyperphosphatemia. However, none of the respective products (bromfenac, colestilan, and tafluprost/timolol) are specifically recommended by guidelines, raising the question whether their unavailability really results in a major risk for public health, as long as appropriate alternatives from the same class with similar product characteristics are available. Furthermore, unavailable medicines that are specifically recommended in clinical guidelines might potentially be substituted by other medicines as long as comparators from the same class with similar product characteristics are available.

In addition to the inconsistencies between G-BA appraisals and guideline recommendations, our review also revealed major heterogeneities across guidelines. In two therapeutic areas (Dupuytren’s contracture and hypolactasia), no guidelines were available at all and the only available guideline covering wound healing and the effect of living larvae of *Lucilia sericata* was developed in 2012 [35]. In contrast, 20 guidelines covered prostate cancer, 9 of which had included specific recommendations for sipuleucel-T in patients with asymptomatic metastatic prostate cancer prior to the withdrawal of the medicine due to bankruptcy of the manufacturer [36]. Heterogeneity between guidelines can partially been explained by different standards of care across various countries. However, in contrast to HTA appraisals that are automatically initiated upon availability of an innovative medicine, clinical guidelines are often characterized by a lack of timely renewal and therefore may not always be up to date with the most recent developments.

A key discussion point in the current German HTA environment is the value of G-BA assessments in shaping treatment algorithms. In contrast to e.g. the UK, where most treatment guidelines are issued by NICE, the German Society of Hematology and Oncology makes an enormous effort to keep clinical guidelines i) under their influence and ii) always up to date. Clinical positioning statements are issued for each of the G-BA appraisals in oncology, and, despite criticism regarding insufficient methodological rigor, the ‘Onkopedia’ guidelines [37] aim towards clinically shaped treatment algorithms, thereby ensuring best clinical practice. A recently conducted comparative review of Onkopedia guidelines and G-BA appraisals revealed that 38% of patient groups established by the G-BA in the appraisal of oncological medicines partially or fully deviated from those mentioned in the Onkopedia guidelines, and 60% of additional benefit decisions by the G-BA showed a partial or complete discordance with the guidelines [38]. This indicates that many medicines might play an important role in a clinically optimized treatment algorithm despite a ‘no additional benefit’ appraisal by the G-BA.

Withdrawal from the market is particularly painful if a high utilization of the medicine had already occurred. The epilepsy treatment perampanel is considered a useful treatment option for patients with drug-resistant disease due to its unique mechanism of action [39]; moreover, the product is one of the few anticonvulsants with an explicit approval for use in adolescents above the age of 12 [15]. At the time of market withdrawal, more than 5.000 patients were receiving the medicine [7]. Also, when Novo Nordisk withdrew its basal insulin analogue insulin degludec from the German market in January 2016, health care professionals were asked to switch the approximately 40.000 patients receiving the product at that time to an alternative insulin [40]. Diabetes experts considered this mandated therapy change to the disadvantage of patients, as insulin degludec offers a unique safety profile and a longer half-life [41]. Commenting on the recent withdrawal of osimertinib, a tyrosine kinase inhibitor for the treatment of non-small lung cancer carrying the T790 M mutation of the epidermal growth factor receptor (EGFR), the German Society for Hematology and Medical Oncology stated that “all parties involved are right but the damage is on the patients” [42, 43].

Interestingly, it has to be noted that there are several medicines that were re-introduced into the market after the initial withdrawal decision by the pharmaceutical manufacturer. For three medicines (bosuitinib, pitavastin, and dapagliflozin) the re-entry occurred within the time frame of our analysis (cut-off June 1st 2016). Also, ataluren, the only orphan medicine to be withdrawn from the market, as well as mirabegron, linaclotide, and perampanel, were reintroduced later on.

Within the German AMNOG environment the decision to withdraw a medicine from the market is taken by the manufacturer. An analysis of the reasons why the involved companies decided to withdraw the medicines (e.g. outcomes of the benefit assessment and/or the subsequent price negotiation), or why some of the medicines were reintroduced at a later time point, was beyond the scope of this analysis.

Our analysis compares HTA appraisals and clinical guidelines only. The determination of the appropriate comparative therapy within the G-BA procedures also includes systematic reviews and Cochrane reviews. Including those additional sources of evidence in this comparative analysis is therefore part of the future research agenda. An in-depth comparison of clinical guidelines, the recommendations for the various treatment lines and the rating systems of the guidelines is part of the upcoming research agenda.

## Conclusions

Our analysis revealed considerable differences across clinical guidelines, as well as between clinical guidelines and HTA appraisals, for the medicines that were withdrawn from the German market. Better alignment of the clinical perspective in the determination of future treatment algorithms and close collaboration between all involved parties (G-BA, IQWiG, physician associations, and patient representatives) is required to achieve and maintain optimization of patient care in an increasingly HTA-shaped clinical environment.

## Additional files

Additional file 1: **Figure S1.** Identification and analysis of guidelines. (DOCX 318 kb)

Additional file 2: **Table S1.** Guideline recommendations for individual medicines and/or classes. (DOCX 75 kb)

## Abbreviations

AMNOG: Act on the Reform of the Market for Medical Products (‘Arzneimittelmarktneuordnungsgesetz’)
EGFR: Epidermal growth factor receptor
EMA: European Medicines Agency
G-BA: Federal Joint Committee (‘Gemeinsamer Bundesausschuss’)
GKV-SV: National Association of the Statutory Health Insurance Funds (‘GKV Spitzenverband’)
HTA: Health Technology assessment
IQWIG: ‘Institut für Qualität und Wirschaftlichkeit im Gesundheitswesen’
NICE: National Institute for Health and Care Excellence

## References

[1] Cerri KH, Knapp M, Fernandez JL (2014). Decision making by NICE: examining the influences of evidence, process and context. Health Econ Policy Law.

[2] Maison P, Zanetti L, Solesse A, Bouvenot G, Massol J, ISPEP group of the French National Authority for Health (2013). The public health benefit of medicines: how it has been assessed in France? The principles and results of five years’experience. Health Policy.

[3] Deutscher Bundestag. [Sozialgesetzbuch (SGB) Fünftes Buch (V) - Gesetzliche Krankenversicherung - (Artikel 1 des Gesetzes v. 20. Dezember 1988, BGBl. I S. 2477, letzte Änderung durch Artikel 3 des Gesetzes vom 20. Dezember 2012 (BGBI. I S. 2781))]. 1988. https://www.gesetze-im-internet.de/bundesrecht/sgb_5/gesamt.pdf. Accessed 15 Sept 2016.

[4] Ruof J, Schwartz FW, Schulenburg JM, Dintsios CM (2014). Early benefit assessment (EBA) in Germany: analysing decisions 18 months after introductin the new AMNOG legislation. Eur J Health Econ.

[5] Ludwig S, Dintsios CM (2016). Arbitration board setting reimbursement amounts for pharmaceutical innovations in Germany when Price negations between payers and manufacturers fail: an empirical analysis of 5 Years' experience. Value Health.

[6] Verband Forschender Arzneimittelhersteller (vfa). [Das AMNOG im vierten Jahr]. 2014. https://www.vfa.de/download/amnog-4tes-jahr-lang.pdf. Accessed 13 Sept 2016.

[7] Deutsche Apotheker Zeitung. [DAV beschwert sich über Fycompa]. 2016. https://www.deutsche-apotheker-zeitung.de/news/artikel/2016/04/28/dav-beschwert-sich-uber-fycompa. Accessed 13 Sept 2016.

[8] Leyens L, Brand A (2016). Early patient access to medicines: health technology assessment bodies need to catch up with new marketing authorization methods. Public Health Genomics.

[9] Kassenärztliche Vereinigung Westfalen-Lippe. [Frühe Nutzenbewertung: Erfahrungen nach vier Jahren]. 2015. https://www.kvwl.de/arzt/verordnung/arzneimittel/info/invo/fruehe_nutzenbewertung_rueckblick_invo.pdf. Accessed 13 Sept 2016.

[10] Greiner W, Witte J, Rebscher H (2016). AMNOG-Report 2016 - Nutzenbewertung von Arzneimitteln in Deutschland.

[11] GKV-Spitzenverband. [Übersicht zu den Erstattungsbetragsverhandlungen nach § 130b SGB V]. 2016. https://www.gkv-spitzenverband.de/krankenversicherung/arzneimittel/verhandlungen_nach_amnog/ebv_130b/ebv_nach_130b.jsp?pageNo=3&submitted=true&sort=&descending=&searchterm=Suchbegriff+eingeben&status=Alle&specialFeature=#arzneimittelliste. Accessed 13 Sept 2016.

[12] IGES Institut GmbH. [Arzneimittel-Atlas]. 2015. http://www.arzneimittel-atlas.de/im-fokus/amnog/versorgung/index_ger.html. Accessed 13 Sept 2016.

[13] Schwabe U, Paffrath D (2015). Arzneiverordnungs-Report.

[14] G-BA. [G-BA legt Kriterien für Bestandsmarktaufruf fest und bestimmt erste Wirkstoffgruppen für die Nutzenbewertung]. 2013. https://www.g-ba.de/institution/presse/pressemitteilungen/485/. Accessed 13 Sept 2016.

[15] G-BA. Overview of products [Übersicht der Wirkstoffe]. 2016. http://www.g-ba.de/informationen/nutzenbewertung/. Accessed 16 Mar 2017.

[16] European Medicines Agency. European public assessment reports. 2016. http://www.ema.europa.eu/ema/index.jsp?curl=pages/medicines/landing/epar_search.jsp&mid=WC0b01ac058001d124. Accessed 13 Sept 2016.

[17] Lauer Fischer GmbH. Lauer-Taxe. 2016. https://www.cgm.com/lauer-fischer/loesungen_lf/lauer_taxe_lf/lauer_taxe.de.jsp. Accessed 13 Sept 2016.

[18] Arbeitsgemeinschaft der Wissenschaftlichen Medizinischen Fachgesellschaften. [Aktuelle Leitlinien]. 2016. https://www.awmf.org/awmf-online-das-portal-der-wissenschaftlichen-medizin/awmf-aktuell.html. Accessed 27 Jan 2017.

[19] European Medicines Agency. Provenge - Withdrawal of the marketing authorisation in the European Union (public statement). 2015. http://www.ema.europa.eu/docs/en_GB/document_library/Public_statement/2015/05/WC500186950.pdf. Accessed 13 Sept 2016.

[20] European Medicines Agency. BindRen - Withdrawal of the marketing authorisation in the European Union (public statement). 2015. http://www.ema.europa.eu/docs/en_GB/document_library/Public_statement/2015/03/WC500184373.pdf. Accessed 13 Sept 2016.

[21] GlaxoSmithKline. GlaxoSmithKline: Advance notification of Trobalt® discontinuation. 2016. https://assets.publishing.service.gov.uk/media/57fe4b6640f0b6713800000c/Trobalt_letter.pdf. Accessed 27 Jan 2017.

[22] G-BA. [Sipuleucel-T - Nutzenbewertung gemäß §35a SGB V - Beschluss]. 2015. https://www.g-ba.de/downloads/40-268-3155/2015-03-19_AM-RL-XII_Sipuleucel-T_2014-10-01-D-139_TrG.pdf. Accessed 15 Sept 2016.

[23] IQWiG. [Orientierende Aufbereitung für das Thema "Kataraktoperation"]. 2009. https://www.iqwig.de/download/V09-01C_Rapid-Report_Orientierende_Aufbereitung_Kataraktoperation.pdf. Accessed 19 Sept 2016.

[24] Deutscher Bundestag. [Entwurf eines Gesetzes zur Neuordnung des Arzneimittelmarktes in der gesetzlichen Krankenversicherung (Arzneimittelmarktneuordnungsgesetz – AMNOG)]. 2010. http://dipbt.bundestag.de/dip21/btd/17/031/1703116.pdf. Accessed 3 Feb 2017.

[25] Cassel D, Ulrich V (2015). AMNOG auf dem ökonomischen Prüfstand - Funktionsweise, Ergebnisse und Reformbedarf der Preisregulierung für neue Arzneimittel in Deutschland.

[26] Schlegl E, Durournau P, Ruof J (2017). Different weights of evidence-based medicine triad in regulatory, health technology assessment, and clinical decision making. Pharm Med.

[27] Staab T, Isbary G, Amelung VE, Ruof J (2016). Inconsistent approaches of the G-BA regarding acceptance of primary study endpoints as being relevant to patients - an analysis of three disease areas: oncological, metabolic, and infectious diseases. BMC Health Serv Res.

[28] National Institute for Health and Care Excellence. Irritable bowel syndrome in adults: diagnosis and management. 2015. https://www.nice.org.uk/guidance/cg61/resources/irritable-bowel-syndrome-in-adults-diagnosis-and-management-975562917829. Accessed 20 Jan 2017. 32073807

[29] Cuchel M, Bruckert E, Ginsberg HN, Raal FJ, Santos RD, Hegele RA (2014). Homozygous familial hypercholesterolaemia: new insights and guidance for clinicians to improved detection and clinical managment. A position paper from the consensus panel on familial Hypercholesterolaemia of the European atherosclerosis society. Eur Heart J.

[30] Hasan A, Falkai P, Wobrock T, Lieberman J, Glenthoj B, Gattaz WF (2012). World Federation of Societies of biological psychiatry (WFSBP) guidelines for biological treatment of schizophrenia, part 1: update 2012 on the acute treatment of schizophrenia and the management of treatment resistance. World J Biol Psychiatry.

[31] Gormley EA, Lightner DJ, Burgio KL, Chai TC, Clemens JQ, Culkin DJ, et al. Diagnosis and Treatment of Overactive Bladder (Non-Neurogenic) in Adults: AUA/SUFU Guideline. 2014. http://www.auanet.org/guidelines/overactive-bladder-(oab)-(aua/sufu-guideline-2012-amended-2014). Accessed 18 Dec 2017. 10.1016/j.juro.2012.09.079 23098785

[32] National Institute for Health and Care Excellence. Epilepsies: diagnosis and management. 2016. https://www.nice.org.uk/guidance/cg137/resources/epilepsies-diagnosis-and-management-35109515407813. Accessed 20 Jan 2017. 32027473

[33] Van Cutsem E, Cervantes A, Adam R, Sobrero A, Van Krieken JH, Aderka D (2016). ESMO consensus guidelines for the management of patients with metastatic colorectal cancer. Ann Oncol.

[34] ESMO/European Sarcoma Network Working Group (2014). Gastrointestinal stromal tumours: ESMO clinical practice guidelines for diagnosis, treatment and follow-up. Ann Oncol.

[35] Deutsche Gesellschaft für Wundheilung und Wundbehandlung. [Lokaltherapie chronischer Wunden bei Patienten mit den Risiken periphere arterielle Verschlusskrankheit, Diabetes mellitus, chronische venöse Insuffizienz]. 2012. https://www.awmf.org/leitlinien/detail/ll/091-001.html. Accessed 20 Jan 2017.

[36] Jaroslawski S, Caban A, Toumi M (2015). Sipuleucel - T (Provenge®): autopsy of an innovative change in paradigm in Cancer treatment. Value Health.

[37] Deutsche Gesellschaft für Hämatologie und medizinische Onkologie (DGHO). Onkopedia guidelines. 2017. https://www.onkopedia-guidelines.info/en/onkopedia/guidelines. Accessed 25 Oct 2017.

[38] Holzerny P, Werner S, Ruof J. Are FJC appraisals suitable to guide therapeutic decisions. Analysis of consistency between clinical guidelines and FJC appraisals in Oncology. Gesundh ökon Qual manag. 2018. 10.1055/s-0043-121590.

[39] Frampton JE (2015). Perampanel: a review in drug-resistant epilepsy. Drugs.

[40] Deutsche Gesellschaft für Endokrinologie. [Insulin Degludec (Tresiba®) gestern von der Amerikanischen Arzneibehörde (FDA) zugelassen – und in Deutschland Ende dieses Monats trotz Zulassung von der Firma wieder vom Markt genommen]. 2015. http://blog.endokrinologie.net/insulin-degludec-trotz-zulassung-vom-markt-genommen-2278/. Accessed 13 Sept 2016.

[41] Gallwitz B. [Für Patienten folgenreich]. 2015. https://www.aerzteblatt.de/pdf/112/43/p17.pdf?ts=20.10.2015+09%3A01%3A51. http://www.diabetologie-online.de/a/1753487. Accessed 13 Sept 2016.

[42] AstraZeneca. TAGRISSO™ (osimertinib) approved in EU as first-in-class treatment for patients with EGFR T790M mutation-positive metastatic non-small cell lung cancer. 2016. https://www.astrazeneca.com/media-centre/press-releases/2016/tagrisso-osimertinib-approved-in-eu-as-first-in-class-treatment-for-lung-cancer-03022016.html. Accessed 16 Mar 2017.

[43] Deutsche Gesellschaft für Hämatologie und medizinische Onkologie (DGHO). [Weiteres neues Krebsmedikament vom Markt genommen: Alle beteiligten Institutionen haben Recht, aber den Schaden haben die Patienten]. 2016. https://www.dgho.de/aktuelles/presse/pressearchiv/2016/weiteres-neues-krebsmedikament-vom-markt-genommen-alle-beteiligten-institutionen-haben-recht-aber-den-schaden-haben-die-patienten. Accessed 14 Nov 2016.

[44] Sauro KM, Wiebe S, Dunkley C, Janszky J, Kumlien E, Moshe S (2016). The current state of epilepsy guidelines: a systematic review. Epilepsia.

