# Supplementary material for: “Market withdrawals” of medicines in Germany after AMNOG: a comparison of HTA ratings and clinical guideline recommendations
Source: Health Econ Rev. 2018 Sep 18;8:23. doi: 10.1186/s13561-018-0209-3 (PMC6755547; doi:10.1186/s13561-018-0209-3)
Supplement: Supplementary file 1 — Figure S1. Identification and analysis of guidelines. (DOCX 318 kb) [file 13561_2018_209_MOESM1_ESM.docx]

# Additional file 1: Figure S1 Identification and analysis of guidelines.


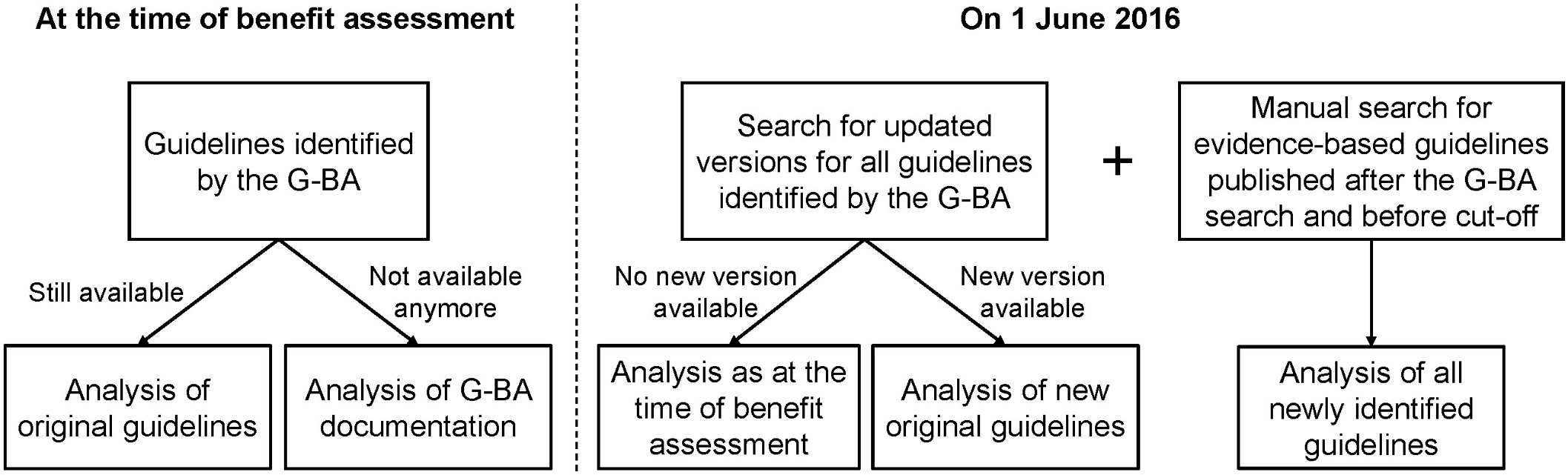


G-BA: Federal Joint Committee.
