# Supplementary material for: “Market withdrawals” of medicines in Germany after AMNOG: a comparison of HTA ratings and clinical guideline recommendations
Source: Health Econ Rev. 2018 Sep 18;8:23. doi: 10.1186/s13561-018-0209-3 (PMC6755547; doi:10.1186/s13561-018-0209-3)
Supplement: Supplementary file 2 — Table S1. Guideline recommendations for individual medicines and/or classes. (DOCX 75 kb) [file 13561_2018_209_MOESM2_ESM.docx]

Additional file 2: Table S1 Guideline recommendations for individual medicines and/or classes.

| **At time of benefit assessment** | | | | **Additional guidelines June 2016** | | | |
| --- | --- | --- | --- | --- | --- | --- | --- |
| **Product (indication) & guideline** | **Evidence rating y/n*** | **Treatment algorithm y/n**** | **Recommen-dation***** | **Guideline** | **Evidence rating y/n*** | **Treatment algorithm y/n**** | **Recommen-dation** |
| **Aliskiren/Amlodipine**  (Hypertension)   - Europe 2009 (1) - NICE CG127 2011 (2) - US 2010_1^†^ - Australia 2008 (3) | No  Yes  n/a  No | Yes  Yes  n/a  Yes | Medicine  No  No  No | - Europe 2013_1 (4) - US 2014_1 (5) | Yes  Yes | Yes  No | Medicine  No |
| **Bromfenac**  (Postoperative management of cataract surgery)   - UK 2010 (6) - Philippines 2005 (7) - US 2011 (8) - Canada 2008_1 (9) - NICE IPG264 2008 (10) - NICE IPG209 2007 (11) - British Columbia 2007 (12) | Yes  Yes  Yes  Yes  No  No  No | No  No  No  No  No  No  No | Class  Class  Class  Class  No  No  No | - US 2013_1 (13) - US 2013_2 (14) | Yes  Yes | No  No | Class  No |
| **Canagliflozin**  (Diabetes)   - Germany S3 2013_1 (15) - Germany 2009 (16) - Scotland 2010^†^ - NICE CG87 2010^†^ - US 2014_2 (17) | Yes  No  n/a  n/a  Yes | Yes  Yes  n/a  n/a  Yes | Class  No  No  No  No | - Germany S3 2014_1 (18) - NICE NG28 2015 (19) - US/Europe 2015 (20) | Yes  Yes  No | Yes  Yes  Yes | Class  Medicine  Medicine |
| **Canagliflozin/Metformin**  (Diabetes)   - Germany S3 2014_2 (21) - Germany 2009 (16) - Scotland 2010^†^ - NICE CG87 2010^†^ - US 2014_2 (17) | Yes  No  n/a  n/a  Yes | Yes  Yes  n/a  n/a  Yes | Class  No  No  No  No | - Germany S3 2014_1 (18) - NICE NG28 2015 (19) - US/Europe 2015 (20) | Yes  Yes  No | Yes  Yes  Yes | Class  Medicine  Medicine |
| **Colestilan**  (Hyperphosphatemia)   - International 2009 (22) | Yes | No | Class | - International 2013 (23) | Yes | No | Class |
| **Gaxilose**  (Hypolactasia)   - No guidelines identified |  |  |  | - No additional guidelines |  |  |  |
| **Insulin degludec**  (Diabetes)   - Germany S3 2016 (15) - German 2009 (16) - Scotland 2010^†^ - NICE CG87 2010^†^ - US 2014_2 (17) - Germany S3 2011_1 (24) - NICE CG15 2004 (25) | Yes  No  n/a  n/a  Yes  Yes  No | Yes  Yes  n/a  n/a  Yes  No  No | Class  Class  Class  Class  Class  Class  Class | - Germany S3 2014_1 (18) - NICE NG28 2015 (19) - US/Europe 2015 (20) - NICE NG18 2015 (26) | Yes  Yes  No  Yes | Yes  Yes  Yes  Yes | Class  Class  Medicine |
| **Living larvae from Lucilia sericata**  (Wound healing)   - Germany S3 2012 (27) | Yes | No | Medicine |  |  |  |  |
| **Linaclotid**  (Irritable bowel syndrome)   - Germany S3 2011_2 (28) - NICE CG61 2008^†^ - Great Britain 2012 (29) - Great Britain 2007_1 (30) | Yes  n/a  Yes  Yes | Yes  n/a  No  No | No  No  No  No | - NICE CG61 2015 (31) | Yes | Yes | Medicine |
| **Linagliptin**  (Diabetes)   - Scotland 2010^†^ - NICE CG87 2010^†^ - Germany S3 2004 (32) | n/a  n/a  Yes | n/a  n/a  Yes | Class  Class  No | - Germany S3 2014_1 (18) - NICE NG 28 2015 (19) - US/Europe 2015 (20) | Yes  Yes  No | Yes  Yes  Yes | Medicine  Class  Medicine |
| **Lixisenatide**  (Diabetes)   - Germany 2013 (33) - Germany S3 2009 (16) - Scotland 2010^†^ - NICE CG87 2010^†^ - US 2012_1 (34) | Yes  No  n/a  n/a  Yes | Yes  Yes  n/a  n/a  No | Class  Class  Class  Class  Class | - US 2014_2 (17) - Germany S3 2014_1 (18) - NICE NG28 2015 (19) - US/Europe 2015 (20) | Yes  Yes  Yes  No | Yes  Yes  Yes  Yes | Class  Medicine  Class  Medicine |
| **Lomitapide**  (Hypercholesterolemia)   - NICE CG71 2008 (35) - Europe 2011_1 (36) | Yes  Yes | Yes  No | No  No | - Europe 2014 (37) - Europe 2016_1 (38) | No  Yes | Yes  Yes | Medicine  Medicine |
| **Lurasidone**  (Schizophrenia)   - International 2012 (39) - Malaysia 2009 (40) - UK 2009 (41) - US 2010_2 (42) | Yes  Yes  No  No | No  Yes  No  No | Medicine  Class  Class  Class | - NICE CG178 2014 (43) - Scotland 2013 (44) | Yes  Yes | Yes  No | Class  Class |
| **Microbial collagenase**  (Dupuytren’s contracture)   - No guidelines identified |  |  |  | - No additional guidelines |  |  |  |
| **Mirabegron**  (Overactive bladder)   - Germany S3 2010 (45) - Europe 2010 (46) - Canada 2012_1 (47) - Europe 2011_2 (48) - Canada 2012_2 (49) - US 2012_2 (50) | Yes  Yes  Yes  Yes  Yes  Yes | Yes  No  Yes  Yes  No  Yes | No  No  No  No  No  No | - US 2014_3 (51) | Yes | No | Medicine |
| **Perampanel**  (Epilepsy)   - NICE CG20 2004 (52) | Yes | Yes | No | - NICE CG137 2012 (53) | Yes | Yes | No |
| **Regorafenib**  (Colorectal carcinoma)   - Germany S3 2008 (54) - Canada 2008_2^†^ - NICE CG131 2001^†^ - Great Britain 2007_2 (55) - US NCCN 2012^†^ - Scotland 2011^†^ - Europe 2010 (56)   (Gastrointestinal stromal tumor)   - No guidelines identified | Yes  n/a  n/a  Yes  n/a  n/a  Yes | No  n/a  n/a  No  n/a  n/a  Yes | No  No  No  No  No  No  No | - US 2016_1 (57) - US 2016_2 (58) - Europe 2016_2 (59) - NICE CG131 2011 (60) - Germany S3 2014_3 (61) - Scotland 2016 (62) - Europe 2014 (63) | Yes  Yes  Yes  Yes  Yes  Yes  Yes | Yes  Yes  Yes  Yes  No  No  No | Medicine  Medicine  Medicine  No  No  No  Medicine |
| **Retigabine**  (Epilepsy)   - NICE CG20 2012 (64) | Yes | Yes | Medicine | - NICE CG137 2012 (53) | Yes | Yes | Medicine |
| **Sipuleucel-T**  (Prostate cancer)   - Europe 2013_2^†^ - US NCCN 2012^†^ - US NCCN 2013^†^ - US AUA 2013 (65) - NICE 2009^†^ - Canada 2010 (66) - Spain SEOM 2012 (67) - Spain SOGUG 2012 (68) - Australia 2010_1 (69) - Europe 2013_3 (70) - Germany S3 2011_3 (71) | n/a  n/a  n/a  Yes  n/a  Yes  No  Yes  Yes  Yes  Yes | n/a  n/a  n/a  No  n/a  Yes  Yes  Yes  No  No  No | Medicine  Medicine  Medicine  Medicine  No  No  No  No  No  No  No | - Germany S3 2014_4 (72) - Europe 2015_1 (73) - Australia 2015 (74) - Europe 2015_2 (75) - US 2016_3 (76) - Canada 2015 (77) - Spain SEOM 2014 (78) - Spain SOGUG 2015 (79) - NICE CG175 2014 (80) | Yes  Yes  Yes  Yes  Yes  Yes  Yes  Yes  Yes | No  No  No  Yes  Yes  Yes  No  No  Yes | Medicine  Medicine  Medicine  Medicine  Medicine  No  No  No  No |
| **Tafluprost/Timolol**  (Glaucoma)   - Australia 2010_2 (81) - NICE CG85 2009 (82) | Yes  Yes | Yes  No | Class  Class |  |  |  |  |
| **Vildagliptin**  (Diabetes)   - Germany S3 2013_2 (33) - German 2009 (16) - Scotland 2010^†^ - NICE CG87 2010^†^ - US 2012_1 (34) | Yes  No  n/a  n/a  Yes | Yes  Yes  n/a  n/a  No | Medicine  Medicine  Medicine  Medicine  Medicine | - US/Europe 2015 (20) - Germany S3 2014_1 (18) - NICE NG28 2015 (19) - US 2014_2 (17) | No  Yes  Yes  Yes | Yes  Yes  Yes  Yes | Medicine  Medicine  Class  Class |
| **Vildagliptin/Metformin**  (Diabetes)   - Germany S3 2013_2 (33) - Germany 2009 (16) - Scotland 2010^†^ - NICE CG87 2010^†^ - US 2012_1 (34) | Yes  No  n/a  n/a  Yes | Yes  Yes  n/a  n/a  No | Medicine  Medicine  Medicine  Medicine  Medicine | - US/Europe 2015 (20) - Germany S3 2014_1 (18) - NICE NG28 2015 (19) - US 2014_2 (17) | No  Yes  Yes  Yes | Yes  Yes  Yes  Yes | Medicine  Medicine  Class  Class |

* Guideline includes the methodology used for evidence rating.

** Guideline includes a graphical display of the suggested treatment algorithm; diagnostic and other graphical algorithms were not considered.

*** 3 scoring options: medicine mentioned and recommended; class recommended; no recommendation.

^†^: The G-BA documentation was analyzed as the guideline is no longer available.

AUA: American Urological Association; n/a: not applicable (e.g. as relevant information not available); NCCN: National Comprehensive Cancer Network; NICE: National Institute for Health and Care Excellence (UK); SEOM: Sociedad Española de Oncología Médica; SOGUG: Spanish Oncology Genitourinary Group.

**References**

1. Mancia G, Laurent S, Agabiti-Rosei E, Ambrosioni E, Burnier M, Caulfield MJ, et al. Reappraisal of European guidelines on hypertension management: a European Society of Hypertension Task Force document. J Hypertens 2009;27:2121-58.
2. National Clinical Guideline Centre. Hypertension. The clinical management of primary hypertension in adults. Clinical Guideline 127. Update of clinical guidelines 18 and 34. 2011. https://www.ncbi.nlm.nih.gov/pubmed/22855971. Accessed 25 Oct 2017.
3. National Heart Foundation of Australia (National Blood Pressure and Vascular Disease Advisory Committee). Guide to management of hypertension 2008. Updated December 2010. 2010. www.heartfoundation.org.au. Accessed 25 Oct 2017.
4. Mancia G, Fagard R, Narkiewicz K, Redon J, Zanchetti A, Böhm M, et al. 2013 ESH/ESC guidelines for the management of arterial hypertension: the Task Force for the Management of Arterial Hypertension of the European Society of Hypertension (ESH) and of the European Society of Cardiology (ESC). Eur Heart J. 2013;34:2159-219.
5. Kenning I, Kerandi H, Luehr D, Margolis K, O’Connor P, Pereira C, et al. Institute for Clinical Systems Improvement. Hypertension Diagnosis and Treatment. Updated November 2014. 2014. https://www.icsi.org/_asset/wjqy4g/HTN.pdf. Accessed 25 Oct 2017.
6. The Royal College of Ophthalmologists. Cataract Surgery Guidelines. 2010. https://www.rcophth.ac.uk/wp-content/uploads/2014/12/2010-SCI-069-Cataract-Surgery-Guidelines-2010-SEPTEMBER-2010.pdf. Accessed 20 Jan 2017.
7. Philippine Academy of Ophthalmology. Clinical Practice Guideline for the Management of Cataract Among Adults. 2005. http://www.pao.org.ph/PDF/pao-member/personal-announcements/clinical%20practice%20guideline%20for%20cataract.pdf. Accessed 20 Jan 2017.
8. American Academy of Ophthalmology. Preferred Practice Pattern: Cataract in the Adult Eye. 2011. http://bdoc.info/dl/informationen/Cataract-in-the-Adult-Eye-2011-AAO-komplett.pdf. Accessed 20 Jan 2017.
9. Canadian Ophthalmological Society. Canadian Ophthalmological Society evidence-based clinical practice guidelines for cataract surgery in the adult eye. 2008. http://www.cos-sco.ca/cpgs/COS_CataractCPGs_Oct08.pdf. Accessed 20 Jan 2017.
10. National Institute of Clinical Excellence. Implantation of multifocal (non-accommodative) intraocular lenses during cataract surgery. Interventional procedure guidance. 2008. http://www.nice.org.uk/guidance/ipg264. Accessed 25 Oct 2017.
11. National Institute of Clinical Excellence. Implantation of accommodating intraocular lenses for cataract. Interventional procedure guidance. 2007. http://www.nice.org.uk/guidance/ipg209. Accessed 25 Oct 2017.
12. British Columbia Medical Association. Guidelines and Protocols. Advisory Committee. Cataract - Treatment of Adults. Effective Date: September 1, 2005; Update 2007. 2007. http://www2.gov.bc.ca/gov/content/health/practitioner-professional-resources/bc-guidelines/cataract. Accessed 25 Oct 2017.
13. American Academy of Ophthalmology. Preferred Practice Pattern Clinical Questions: Uveitis and Cataract Surgery. 2013. https://www.aao.org/clinical-questions/uveitis-cataract-surgery. Accessed 20 Jan 2017.
14. American Academy of Ophthalmology. Summary Benchmark for Preferred Practice Pattern Clinical Questions: Uveitis and Cataract Surgery. 2013. https://www.aao.org/clinical-questions/uveitis-cataract-surgery. Accessed 20 Jan 2017.
15. Programm für Nationale VersorgungsLeitlinien. [Nationale VersorgungsLeitlinie: Therapie des Typ-2-Diabetes. Langfassung, 1. Auflage, Version 2]. 2013. http://www.leitlinien.de/mdb/downloads/nvl/diabetes-mellitus/archiv/therapie/dm-therapie-1aufl-vers2-lang.pdf. Accessed 20 Jan 2017.
16. Arzneimittelkommission der deutschen Ärzteschaft. [Diabetes mellitus. Empfehlungen zur antihyperglykämischen Therapie des Diabetes melllitus Typ 2. 2. Auflage 2009]. 2009. https://www.akdae.de/Arzneimitteltherapie/TE/A-Z/PDF/Diabetes2.pdf#page=1&view=fitB. Accessed 25 Oct 2017.
17. American Diabetes Association. Standards of Medical Care in Diabetes - 2014. Diabetes Care. 2014;37 Suppl 1:S14-80.
18. Programm für Nationale VersorgungsLeitlinien. [Nationale VersorgungsLeitlinie: Therapie des Typ-2-Diabetes. Langfassung, 1. Auflage, Version 4]. 2014. http://www.leitlinien.de/mdb/downloads/nvl/diabetes-mellitus/dm-therapie-1aufl-vers4-lang.pdf. Accessed 20 Jan 2017.
19. National Institute for Health and Care Excellence. Type 2 diabetes in adults: management. Nice Guideline 28. 2015. https://www.nice.org.uk/guidance/ng28/resources/type-2-diabetes-in-adults-management-1837338615493. Accessed 20 Jan 2017.
20. Inzucchi SE, Bergenstal RM, Buse JB, Diamant M, Ferrannini E, Nauck M, et al. Management of hyperglycaemia in type 2 diabetes, 2015: a patient-centred approach. Update to a position statement of the American Diabetes Association and the European Association for the Study of Diabetes. Diabetologia. 2015;58:429-42.
21. Programm für Nationale VersorgungsLeitlinien. [Nationale VersorgungsLeitlinie: Therapie des Typ-2-Diabetes. Langfassung, 1. Auflage, Version 3]. 2014. http://www.leitlinien.de/mdb/downloads/nvl/diabetes-mellitus/archiv/therapie/dm-therapie-1aufl-vers3-lang.pdf. Accessed 20 Jan 2017.
22. Kidney Disease: Improving Global Outcomes CKDMBDWG. KDIGO clinical practice guideline for the diagnosis, evaluation, prevention, and treatment of Chronic Kidney Disease-Mineral and Bone Disorder (CKD-MBD). Kidney Int Suppl. 2009:S1-130.
23. Kidney Disease: Improving Global Outcomes (KDIGO) CKD Work Group. KDIGO 2012 Clinical Practice Guideline for the Evaluation and Management of Chronic Kidney Disease. Kidney Int Suppl. 2013;3:136-50.
24. Böhm BO, Dreyer M, Fritsche A, Füchtenbusch M, Gölz S, Martin S. [S3-Leitlinie Therapie des Typ-1-Diabetes]. 2011. http://www.deutsche-diabetes-gesellschaft.de/fileadmin/Redakteur/Leitlinien/Evidenzbasierte_Leitlinien/AktualisierungTherapieTyp1Diabetes_1_20120319_TL.pdf. Accessed 20 Jan 2017.
25. National Institute for Health and Care Excellence. Diagnosis and management of type 1 diabetes in children, young people and adults. 2004. https://www.nice.org.uk/guidance/cg15. Accessed 25 Oct 2017.
26. National Institute for Health and Care Excellence. Diabetes (type 1 and type 2) in children and young people: diagnosis and management. 2015. https://www.nice.org.uk/guidance/ng18/resources/diabetes-type-1-and-type-2-in-children-and-young-people-diagnosis-and-management-1837278149317. Accessed 20 Jan 2017.
27. Deutsche Gesellschaft für Wundheilung und Wundbehandlung. [Lokaltherapie chronischer Wunden bei Patienten mit den Risiken periphere arterielle Verschlusskrankheit, Diabetes mellitus, chronische venöse Insuffizienz]. 2012. http://www.awmf.org/uploads/tx_szleitlinien/091-001m_S3_Lokaltherapie_chronischer_Wunden_2012-verlaengert.pdf. Accessed 20 Jan 2017.
28. Layer P, Andresen V, Pehl C, Allescher H, Bischoff SC, Classen M, et al. Irritable Bowel Syndrome: German Consensus Guidelines on Definition, Pathophysiology and Management. AWMF Registriernummer: 021/016. Z Gastroenterol. 2011;49:237-93.
29. McKenzie YA, Alder A, Anderson W, Wills A, Goddard L, Gulia P, et al. on behalf of Gastroenterology Specialist Group of the British Dietetic Association. British Dietetic Association evidence-based practice guidelines for the dietary management of irritable bowel syndrome in adults. J Hum Nutr Diet. 2012;25:260-74.
30. Spiller R, Aziz Q, Creed F, Emmanuel A, Houghton L, Hungin P, et al. Guidelines on the irritable bowel syndrome: mechanisms and practical management. Gut. 2007;56:1770-98.
31. National Institute for Health and Care Excellence. Irritable bowel syndrome in adults: diagnosis and management. 2015. https://www.nice.org.uk/guidance/cg61/resources/irritable-bowel-syndrome-in-adults-diagnosis-and-management-975562917829. Accessed 20 Jan 2017.
32. AWMF. [Nationale Versorgungs-Leitlinie. Diabetes mellitus Typ 2. Version 1.0. vom 10.01.2004]. 2004. http://www.leitlinien.de/mdb/downloads/nvl/diabetes-mellitus/archiv/diabetes/dm2-1-aufl-lang-1.0.pdf. Accessed 25 Oct 2017.
33. Programm für Nationale VersorgungsLeitlinien. [Nationale VersorgungsLeitlinie: Therapie des Typ-2-Diabetes, Kurzfassung, Version 1.0]. 2013. http://www.leitlinien.de/mdb/downloads/nvl/diabetes-mellitus/archiv/therapie/dm-therapie-vers1.0-kurz.pdf. Accessed 20 Jan 2017.
34. American Diabetes Association (ADA). Standards of medical care in diabetes--2012. Diabetes Care. 2012;35 Suppl 1:S11-63.
35. National Institute for Health and Care Excellence. Familial hypercholesterolaemia: identification and management. Clinical guideline 71. 2008. http://www.nice.org.uk/guidance/cg71. Accessed 25 Oct 2017.
36. Reiner Zeljko, Catapano AL, DeBacker G, Graham I, Taskinen MR, Wiklund O, et al. for The Taskforce for the management of dyslipidaemias of the European Society of Cardiology (ESC) and the European Atherosclerosis Society (EAS). ESC/EASD Guidelines for the management of dyslipidaemias. Eur Heart J. 2011;32:1769-818.
37. Cuchel M, Bruckert E, Ginsberg HN, Raal FJ, Santos RD, Hegele RA, et al. Homozygous familial hypercholesterolaemia: new insights and guidance for clinicians to improved detection and clinical managment. A position paper from the Consensus Panel on Familial Hypercholesterolaemia of the European Atherosclerosis Society. Eur Heart J. 2014;35:2146-57.
38. Catapano AL, Graham I, DeBacker G, Wiklund O, Chapman MJ, Drexel H, et al. for The Taskforce for the management of dyslipidaemias of the European Society of Cardiology (ESC) and the European Atherosclerosis Society (EAS). 2016 ESC/EAS Guidelines for the management of dyslipidaemias. Eur Heart J. 2016;37:2999-3058.
39. Hasan A, Falkai P, Wobrock T, Lieberman J, Glenthoj B, Gattaz WF, et al. World Federation of Societies of Biological Psychiatry (WFSBP) Guidelines for Biological Treatment of Schizophrenia, part 1: update 2012 on the acute treatment of schizophrenia and the management of treatment resistance. World J Biol Psychiatry. 2012;13:318-78.
40. Ministry of Health Malaysia. Management of schizophrenia in adults. 2009. http://www.psychiatry-malaysia.org/file_dir/11326515294f66b3f13960b.pdf. Accessed 20 Jan 2017.
41. National Collaborating Centre for Mental Health. Schizophrenia: Core interventions in the treatment and management of schizophrenia in primary and secondary care (update). 2009. https://www.ncbi.nlm.nih.gov/books/NBK11681/pdf/Bookshelf_NBK11681.pdf. Accessed 20 Jan 2017.
42. Buchanan RW, Kreyenbuhl J, Kelly DL, Noel JM, Boggs DL, Fischer BA, et al. The 2009 schizophrenia PORT psychopharmacological treatment recommendations and summary statements. Schizophr Bull. 2010;36:71-93.
43. National Institute for Health and Care Excellence. Psychosis and schizophrenia in adults: prevention and management. 2014. https://www.nice.org.uk/guidance/cg178/resources/psychosis-and-schizophrenia-in-adults-prevention-and-management-35109758952133. Accessed 25 Oct 2017.
44. Scottish Intercollegiate Guidelines Network. Management of schizophrenia. 2013. http://www.sign.ac.uk/pdf/sign131.pdf. Accessed 20 Jan 2017.
45. Deutsche Gesellschaft für Gynäkologie und Geburtshilfe e.V.. [Die überaktive Blase. AWMF Register Nummber 015/007. Stand Juni 2010]. 2010. http://www.agub.de/fileadmin/media/downloads/2010/015-007l_S2k_Ueberaktive_Blase.pdf. Accessed 25 Oct 2017.
46. Oelke M, Bachmann A, Descazeaud A, Emberton M, Gravas S, Michel MC, et al. European Association of Urology. Guidelines on Conservative Treatment of Non-neurogenic Male LUTS. 2010. https://pdfs.semanticscholar.org/a73e/adec182cbb5a74220446fff59a86a86cdb17.pdf. Accessed 25 Oct 2017.
47. Bettez M, Tu LM, Carlson K, Corcos J, Gajewski J, Jolivet M, et al. 2012 Update: Guidelines for Adult Urinary Incontinence Collaborative Consensus Document for the Canadian Urological Association. Can Urol Assoc J. 2012;6:354-63.
48. Thüroff JW, Abrams P, Andersson KE, Artibani W, Chapple CR, Drake MJ, et al. EAU Guidelines on Urinary Incontinence. Eur Urol. 2011;59:387-400.
49. Geoffrion R, Lovatsis D, Walter JE, Chou Q, Easton W, Epp A, et al. Treatments for Overactive Bladder: Focus on Pharmacotherapy. J Obstet Gynaecol Can. 2012;34:1092-101.
50. Gormley EA, Lightner DJ, Burgio KL, Chai TC, Clemens JQ, Culkin DJ, et al. Diagnosis and Treatment of Overactive Bladder (Non-Neurogenic) in Adults: AUA/SUFU Guideline. J Urol. 2012;188:2455-63.
51. Gormley EA, Lightner DJ, Burgio KL, Chai TC, Clemens JQ, Culkin DJ, et al. Diagnosis and Treatment of Overactive Bladder (Non-Neurogenic) in Adults: AUA/SUFU Guideline. 2014. http://www.auanet.org/guidelines/overactive-bladder-(oab)-(aua/sufu-guideline-2012-amended-2014). Accessed 18 Dec 2017.
52. National Institute for Health and Care Excellence. Clinical Guideline 20. The epilepsies: The diagnosis and management of the epilepsies in adults and children in primary and secondary care. 2004. https://www.nice.org.uk/guidance/cg20. Accessed 25 Oct 2017.
53. National Institute for Health and Care Excellence. Epilepsies: diagnosis and management. 2016. https://www.nice.org.uk/guidance/cg137/resources/epilepsies-diagnosis-and-management-35109515407813. Accessed 20 Jan 2017. *Note:* *This is an updated version but the updates did not affect the relevant content; see https://www.nice.org.uk/guidance/cg137.*
54. Schmiegel W, Pox C, Reinacher-Schick A, Arnold D, Graeven U, Heinemann V, et al. S3-Guideline Colorectal Cancer 2004/2008. Z Gastroenterol. 2008;46:1-73.
55. The Association of Coloproctology of Great Britain and Ireland. Guidelines for the Management of Colorectal Cancer. 3^rd^ edition. 2007. https://www.acpgbi.org.uk/content/uploads/2007-CC-Management-Guidelines.pdf. Accessed 25 Oct 2017.
56. Van Cutsem E, Nordlinger B, Cervantes A on behalf of the ESMO Guidelines Working Group. Advanced colorectal cancer: ESMO Clinical Practice Guidelines for treatment. Ann Oncol. 2010;21 Suppl 5:v93-7.
57. National Comprehensive Cancer Network. Colon cancer, version 2.2016. 2016. https://www.tri-kobe.org/nccn/guideline/colorectal/english/colon.pdf. Accessed 20 Jan 2017.
58. National Comprehensive Cancer Network. Rectal cancer, version 2.2016. 2016. https://www.tri-kobe.org/nccn/guideline/colorectal/english/rectal.pdf. Accessed 20 Jan 2017.
59. Van Cutsem E, Cervantes A, Adam R, Sobrero A, Van Krieken JH, Aderka D, et al. ESMO consensus guidelines for the management of patients with metastatic colorectal cancer. Ann Oncol. 2016;27:1386-422.
60. National Institute for Health and Care Excellence. Colorectal cancer: diagnosis and management. Clinical guideline 131. 2011. http://www.nice.org.uk/guidance/cg131. Accessed 25 Oct 2017.
61. Leitlinienprogramm Onkologie (Deutsche Krebsgesellschaft, Deutsche Krebshilfe, AWMF). [S3-Leitlinie Kolorektales Karzinom; Langversion 1.1; AMWF-Registernummer: 021/007OL]. 2014. http://www.awmf.org/uploads/tx_szleitlinien/021-007OLl_S3_KRK_2014-08-verlaengert.pdf. Accessed 25 Oct 2017.
62. Scottish Intercollegiate Guidelines Network. Diagnosis and management of colorectal cancer. A national clinical guideline. SIGN 126. 2016. http://www.sign.ac.uk/sign-126-diagnosis-and-management-of-colorectal-cancer.html. Accessed 25 Oct 2017.
63. ESMO/European Sarcoma Network Working Group. Gastrointestinal stromal tumours: ESMO Clinical Practice Guidelines for diagnosis, treatment and follow-up. Ann Oncol. 2014;25 Suppl 3:iii21-6.
64. National Institute for Health and Care Excellence. The Epilepsies: The diagnosis and management of the epilepsies in adults and children in primary and secondary care. 2012. https://www.ncbi.nlm.nih.gov/pubmedhealth/PMH0068980/pdf/PubMedHealth_PMH0068980.pdf. Accessed 20 Jan 2017.
65. Cookson MS, Roth BJ, Dahm P, Engstrom C, Freedland SJ, Hussain M, et al. Castration-resistant prostate cancer: AUA Guideline. J Urol. 2013;190:429-38.
66. Saad F, Hotte SJ on behalf of the Canadian Urologic Oncology Group and the Canadian Urological Association. Guidelines for the management of castrate-resistant prostate cancer. Can Urol Assoc J. 2010;4:380-4.
67. Arija JAA, Espinosa JC, Duran MAC, Herrero FR. SEOM clinical guidelines for treatment of prostate cancer. Clin Transl Oncol. 2012;14:520-7.
68. Climent MA, Piulats JM, Sanches-Hernandez A. Recommendations from the Spanish Oncology Genitourinary Group for the treatment of patients with metastatic castration-resistant prostate cancer. Crit Rev Oncol Hematol. 2012;83:341-52.
69. Cancer Council Australia. Clinical Practice Guidelines for the management of locally advanced and metastatic prostate cancer. 2010. http://www.cancer.org.au/content/pdf/HealthProfessionals/ClinicalGuidelines/Advanced-Prostate-Cancer-Guidelines-MAR10.pdf. Accessed 25 Oct 2017.
70. Horwich A, Parker C, de Reijke T, Kataja V on behalf of the ESMO Guidelines Working Group. Prostate Cancer: ESMO Clinical Practice Guidelines for diagnosis, treatment and follow-up. Ann Oncol. 2013;24 Suppl 6:vi106-14.
71. Deutsche Gesellschaft für Urologie. [Interdisziplinäre Leitlinie der Qualität S3 zur Früherkennung, Diagnose und Therapie der verschiedenen Stadien des Prostatakarzinoms. Version 1.03; AWMF - Register Nr. 043-022OL]. 2011. http://www.medical-tribune.de/fileadmin/PDF/S3_Leitlinie_Prostatakarzinom_Langfassung_V1-03.pdf. Accessed 25 Oct 2017.
72. Deutsche Gesellschaft für Urologie. Leitlinienprogramm Onkologie. [Interdisziplinäre Leitlinie der Qualität S3 zur Früherkennung, Diagnose und Therapie der verschiedenen Stadien des Prostatakarzinoms]. 2014. http://leitlinienprogramm-onkologie.de/uploads/tx_sbdownloader/LL_Prostata_Langversion_4.0.pdf. Accessed 20 Jan 2017.
73. Parker C, Gillessen S, Heidenreich A, Horwich A, Committee EG. Cancer of the prostate: ESMO Clinical Practice Guidelines for diagnosis, treatment and follow-up. Ann Oncol. 2015;26 Suppl 5:v69-77.
74. Cookson MS, Roth BJ, Dahm P, Engstrom C, Freedland SJ, Hussain M, et al. Castration-Resistant Prostate Cancer: AUA Guideline 2015. 2015. http://www.auanet.org/guidelines/castration-resistant-prostate-cancer-(2013-amended-2015). Accessed 18 Dec 2017.
75. Mottet N, Bellmunt J, Briers E, van den Bergh RCN, Bolla M, van Casteren NJ, et al. Guidelines on Prostate Cancer. 2015. https://uroweb.org/wp-content/uploads/09-Prostate-Cancer_LR.pdf. Accessed 20 Jan 2017.
76. National Comprehensive Cancer Network. Prostate Cancer, version 3.2016. 2016. http://www.tri-kobe.org/nccn/guideline/urological/english/prostate.pdf. Accessed 20 Jan 2017.
77. Saad F, Chi KN, Finelli A, Hotte SJ, Izawa J, Kapoor A, et al. The 2015 CUA-CUOG Guidelines for the management of castration-resistant prostate cancer (CRPC). Can Urol Assoc J. 2015;9:90-6.
78. Cassinello J, Climent MA, Gonzalez del Alba A, Mellado B, Virizuela JA. SEOM Clinical Guidelines for the treatment of metastatic prostate cancer. Clin Transl Oncol. 2014;16:1060-6.
79. Climent MA, Leon-Mateos L, Gonzalez del Alba A, Perez-Valderrama B, Mendez-Vidal MJ, Mellado B, et al. Updated recommendations from the Spanish Oncology Genitourinary Group for the treamtent of patients with metastatic castration-resistant prostate cancer. Crit Rev Oncol Hematol. 2015;96:308-18.
80. National Institute for Health and Care Excellence. Prostate cancer: diagnosis and management. Clinical Guideline 175. 2014. http://www.nice.org.uk/guidance/cg175. Accessed 25 Oct 2017.
81. National Health and Medical Research Council. NHMRC Guidelines for the Screening, Prognosis, Diagnosis, Management and Prevention of Glaucoma 2010. 2010. https://www.nhmrc.gov.au/_files_nhmrc/publications/attachments/cp113_glaucoma_120404.pdf. Accessed 20 Jan 2017.
82. National Institute for Health and Care Excellence. Glaucoma: diagnosis and management. 2009. http://nwlloc.com/guidelines/CG85NICEGuideline.pdf. Accessed 18 Dec 2017.
